# Brain arousal regulation and depressive symptomatology in adults with attention-deficit/hyperactivity disorder (ADHD)

**DOI:** 10.1186/s12868-019-0526-4

**Published:** 2019-08-20

**Authors:** Jue Huang, Christine Ulke, Maria Strauss

**Affiliations:** Department of Psychiatry and Psychotherapy, University of Leipzig, Semmelweisstrasse 10, 04103 Leipzig, Germany

**Keywords:** ADHD, Absolute EEG power, Arousal stability score, Brain arousal regulation, Depression

## Abstract

**Background:**

The aim of the present study was to evaluate the stability of brain arousal in adult attention-deficit/hyperactivity disorder (ADHD) outpatients with and without depressive symptomatology, and its association with depressive symptom severity and absolute electroencephalogram (EEG) power in different frequency bands.

**Methods:**

We included 31 outpatient adults (45.16% females), who were diagnosed according to DSM-IV and received no medication. Their arousal stability score (index of the steepness of arousal decline during a 15-min EEG under resting conditions), the absolute EEG power and self-reports, including depressive and ADHD-related symptoms, were analyzed. Participants were split into an unstable and stable arousal group based on the median (= 6) of the arousal stability score.

**Results:**

ADHD patients in the stable group reported more severe depressive symptoms (*p *= 0.018) and showed reduced absolute EEG power in the delta (0.002 ≤ *p* ≤ 0.025) and theta (0.011 ≤ *p* ≤ 0.034) bands compared to those in the unstable group. There was no correlation between the arousal stability score and self-report-scales concerning ADHD-related symptoms (0.214 ≤ *p* ≤ 0.989), but a positive association with self-reported depressive severity (*p *= 0.018) and negative association with powers in the EEG delta and theta bands (0.001 ≤ *p* ≤ 0.033).

**Conclusions:**

In view of high comorbidity of depression and ADHD in adult patients, these findings support the assumption that brain arousal regulation could be considered as a helpful marker for the clinical differentiation between ADHD and depression.

## Background

Attention-deficit/hyperactivity disorder (ADHD) is a psychiatric disorder with childhood-onset, characterized by symptoms of inattention, emotional instability and/or hyperactivity/impulsivity, affecting daily functioning in at least two life areas. It persists into adulthood with pooled prevalence of 2.5% [1]. ADHD is also commonly associated with different comorbidities, particularly major depression disorder (MDD). A recent 1-year follow-up study reported that the rate of comorbid MDD in adult ADHD patients may be as high as 92.9%, based on a German sample [2]. Given the high prevalence of depressive comorbidity, the differential diagnosis might be challenging for clinicians. Furthermore, ADHD with depressive symptoms is associated with higher demands for previous mental health care, and it may be accompanied more frequently by generalized anxiety disorder and social phobia [3]. Treatment approaches should therefore depend on diagnostic assessment [4, 5], and existing severe mental health disorders, e.g. depression [6, 7]. All these findings highlight the necessity of improved diagnosis. Until now, objective markers, which could improve differential diagnosis, are missing in standard clinical care.

It is commonly accepted that an individual with ADHD is hypoaroused. This concept was firstly described by Satterfield and Dawson [8] illustrating a lower skin conductance level in ADHD patients relative to a healthy control group under resting conditions, and subsequently supported by the following empirical studies [9–13].

Electroencephalography (EEG) is another objective measurement for the electric activity of the brain. There is increasing interest using EEG-measures as diagnostic tools in ADHD patients [14]. Several EEG studies have also reported hypoarousal in ADHD in obtaining elevated slow brain activities, especially theta activity, and elevated beta activity or theta-beta ratio [14, 15]. However, more recent studies failed to replicate previous findings when other comorbidities in ADHD patients were considered. Kim et al. [16] identified decreased absolute theta power in ADHD with problematic internet use as compared to pure ADHD, but not between ADHD with depressive symptoms and other groups. Loo et al. [17] suggested the mediating effects of comorbidities on the quantitative EEG based on their opposite findings to the most consistent EEG finding in ADHD, i.e. lessened instead of elevated theta beta ratio in ADHD patients. The depressive symptomology might also draw effects on EEG outcomes. So far, the important issue of comorbid depressive symptoms in ADHD has not been adequately addressed and remains controversial.

As mentioned above, the hypoarousal in ADHD has well been documents for many years not only upon skin conductance level but also EEG. In this context, the brain arousal regulation model [18, 19] has been developed and picked up in this paper.

The term *brain arousal* in this paper is used as synonym for cortical arousal. It refers to a dimension of functional brain states comprising different levels of wakefulness and sleep [20–22]. The regulation of brain arousal denotes its adaptation to situational requirements. For example, arousal must be increased in case of potential danger, maintained during cognitive task or reduced at bed time. The dysregulations of brain arousal, (i.e. rapid downregulation of arousal to a low level, termed as unstable arousal regulation; or the lack of downregulation, termed as hyperstable arousal regulation) during the EEG recording period (usually 15–20 min), may correspond to certain psychiatric disorders. Recently, unstable arousal regulation over a short period of time in adult ADHD is determined in an empirical EEG study [23]. Therein, the hyperactivity and sensation seeking in ADHD is interpreted as autoregulatory behavior attempting to stabilize unstable arousal regulation. Conversely, a hyperstable brain arousal regulation was demonstrated and replicated in patients with MDD [24–26]. The EEG-related construct of brain arousal has been has been examined as diagnostic and/or predictive marker in patients suffering from ADHD [23] or MDD [24–27]. So far the relationship between the regulation of brain arousal and the depressive symptom has not been studied in ADHD adults.

The locus coeruleus-norepinephrine (LC-NE) system plays a critical role in the modulation of alterations in neuronal activity that are reflected in EEG signals [28–30] permitting the assumption of a relationship between the LC-NE system and the impairment in attention and behavioral control in ADHD patients, as evidenced in several studies [31–33]. Further, animal models provided direct evidence for the involvement of the LC-NE system in ADHD-associated behaviors [34]. In a recent review, ADHD is hypothesized as trait hypoarousal resulting from decreased tonic activity of the LC-NE system [35]. In another review, hyperarousal symptoms can be manifested at children with co-occurred ADHD and posttraumatic stress disorder [36], which could be attributed to the hyperaroused LC-NE system. Thus, considering the overlapped symptoms between MDD and posttraumatic stress disorder liking emotional disturbance and avoidance behaviors, together with the ambiguous findings regarding EEG measurements, especially when the comorbidity in ADHD patients is considered and the empirical findings in the framework of brain arousal regulation model, we suppose that there might also patients showing (hyper)stable arousal regulation in adult ADHD, relating to depressive symptomology.

The level and the regulation of brain arousal can be objectively assessed by a freely downloadable EEG-based algorithm, the Vigilance Algorithm Leipzig (VIGALL 2.1) [37], which has been widely validated [20–22, 38–41]. VIGALL attributes automatically one out of seven EEG-vigilance stages—indexing different level of brain arousal (see Table 1)—to each 1-s EEG segment across entire EEG period, the regulation of brain arousal is illustrated by arousal stability score [38], describing temporal dynamics of EEG-vigilance stages and steepness of brain arousal decline in each individual over a set EEG recording period in a resting condition without any mental and motor task. All subjects were either assigned to unstable or stable arousal regulation group depending on the arousal stability score.

**Table 1:** EEG description for EEG-vigilance stage and operational definition of arousal stability score and assessment criteria

EEG-vigilance stage	EEG description	Arousal stability score	Operational definition	Criterion
0	Low amplitude, desynchronized non-alpha EEG without horizontal SEM	11	Unique occurrence of 0 and A1	More than 2/3 of all segments are attributed as 0/A1 or 0/A stages
		10	Unique occurrence of 0 and A	
A1	Occipital dominant alpha	9	Stage B1 in third 5 min	At least 1/3 of all segments are attributed as stage B1
A2	Shift of alpha to central and frontal areas	8	Stage B1 in second 5 min	
A3	Continued frontalization of alpha	7	Stage B1 in first 5 min	
B1	Low amplitude, desynchronized EEG with horizontal SEM	6	Stage B2/3 in third 5 min	At least 1/3 of all segments are attributed as stage B2/3
		5	Stage B2/3 in second 5 min	
B2/3	Dominant delta and theta	4	Stage B2/3 in first 5 min	
C	Occurrence of K-complex and sleep spindles	3	Stage C in third 5 min	Occurrence of stage C
		2	Stage C in second 5 min	
		1	Stage C in first 5 min	

*SEM* slow eye movement

Moreover, we also aimed to test differences in ADHD and depressive symptoms between groups with unstable and stable brain arousal. Additionally, absolute EEG power based on the entire resting EEG in different frequency bands was analyzed and compared between groups.

## Methods

### Subjects

The sample consisted of 31 unmedicated and drug-free adult outpatients (14 females) recruited at the ADHD outpatient clinic of the University Hospital in Leipzig between the ages of 19 and 48 (mean age = 33.42, SD = 7.06). All ADHD patients had been carefully diagnosed by ADHD-experienced psychiatrists and psychologists according to the diagnostic and statistical manual of mental disorder 4th edition (DSM-IV) and had no known physical or neurological diseases. All patients gave their written informed consent. The protocol was reviewed and approved by the local ethics committee of the University of Leipzig (199-13-15072013). This research was conducted in accordance with the Helsinki Declaration as revised 1989.

### Measurements

For the purpose of subjective evaluation of ADHD-related symptoms, a set of measurements was used during the patient visits at the outpatient clinic: German version of *Conners’ Adults ADHD rating scale*-*self: long version* (CAARS-S:L) [42] containing 66 Items in 8 subscales providing a comprehensive evaluation of the presence and severity of ADHD symptoms. Items from the three subscales directly in accordance with DSM-IV criteria were further included in analyses of this study: DSM-inattention (DSM-IA), DSM-hyperactivity/impulsivity (DSM-HYI) and DSM-global (DSM-G). The items from the other 5 subscales are associated to ADHD symptoms, however, not specifically defined in DSM-IV criteria and were thus excluded. Age- and gender-based standardized T-scores resulting from these subscales were calculated and used in this study. A T-score over 70 indicates a high clinical significance of the respective symptom. Moreover, the German version of *Adult ADHD self*-*report scale* (ASRS-v1.1) [43] symptom checklist with totally 18 items in part A (6 items) and B (12 items) were utilized, which are consistent with DSM-IV criteria and provide an insight into the main symptoms. The frequencies in each part and its total value based on statements from 0 (never) to 4 (very frequently) are reported in this study. The *Wender Utah rating scale* was used for the retrospective diagnosis of ADHD in childhood. The German short version with 25 items (WURS-K) [44] was applied, the sum scores from 21 items evaluated on a five-point scale each are reported in this study. Depressive symptom severity was assessed by the *Beck Depression Inventory*-*II* (BDI-II) [45] composed of 21 questions. A sum score over the cutoff of 13 indicates the existence of mild depressive symptoms.

### EEG recording and pre-processing

EEG was recorded in a dimmed and sound attenuated room. The room temperature was maintained at around 25 degrees Celsius. At the beginning of the recording all participants were placed in a semi-supine position. An eyes-open-close-task followed by a mental calculation task was conducted to confirm that all participants had the same baseline level of wakefulness. Thereafter, a resting EEG in eyes-closed condition was collected for 15 min. The participants were instructed to follow their natural course of brain arousal; they were explicitly allowed to fall asleep. The EEG was recorded with Ag/AgCl electrodes using a QuickAmp amplifier (Brain Products GmbH, Gilching, Germany) from 31 electrode positions according to the extended international 10–20 system using EasyCap (EASYCAP Brain Products GmbH, Gilching, Germany). Each EEG channel was referenced to common average. Impedance of electrodes was kept below 10 kΩ. Additionally, horizontal and vertical eye movements were monitored by two bipolar electrodes.

The EEG signal was analyzed in Brain Vision Analyzer Software Version 2.1 (Brain Products GmbH, Gilching, Germany) by setting the filtering bandpass between 0.5 and 70 Hz (Notch filter 50 Hz). The 15-min EEG data was subdivided into continuous 1-s segments. Muscle or electrical artefacts were manually marked and thus not taken into account in further analysis. Afterwards, an independent component analysis (ICA) was run to remove well-defined sources of artefacts, for example eye movements, cardiogenic artefacts and continuous muscle artefacts. Technical or sweating artefacts were also excluded when present. Graphoelements for sleep-onset (i.e. sleep spindle and K-complex) were identified and tagged by an experienced medical laboratory assistant.

### Assessment of arousal stability score

The arousal stability score quantifies the steepness in decline of brain arousal in each participant. It was assessed by VIGALL 2.1 (https://github.com/danielboettger/VIGALL) as follows: VIGALL attributes—based on the frequency bands with Fast Fourier Transformation (FFT) and source localization with Low Resolution Electromagnetic Tomography (LORETA), one out of seven EEG-vigilance stages ranging from 0 (indicating cognitively active wakefulness), across A1, A2, A3 (indicating relaxed wakefulness), B1 and B2/3 (indicating drowsiness) to C (indicating sleep-onset) to each 1-s EEG segment. These scores for EEG-vigilance stages were used to assess mean EEG-vigilance level (see below). Upon the scoring of each 1-s segment, epochs of 60 s duration with forward moving steps of 1 s (that is, 1–60 s, 2–61 s…) were analyzed for conditions described in Table 1. If one of the conditions was fulfilled, the corresponding stability score was given to this participant.

### Assessment of mean EEG-vigilance level

As aforementioned, mean EEG-vigilance level was indicated by averaging all vigilance scores in a 3-min time block. This was aiming to describe different trends of mean vigilance level with time in different groups.

### EEG power-spectrum analysis

In preparation of the power-spectrum analysis, the EEG was down-sampled to 256 Hz. An automatic artefact rejection function in Brain Vision Analyzer (Brain Products GmbH, Gilching, Germany) was applied additionally to reduce the possible threshold change upon voltage step gradient that were not corrected by the ICA. The power-spectrum analysis was performed via installed FFT in Brain Vision Analyzer (Brain Products GmbH, Gilching, Germany). The FFT converted 1-s time windows with Hanning window function and yielded a resolution of 0.5 Hz into four frequency bands: delta (1–3 Hz), theta (4–7 Hz), alpha (8–12 Hz) and beta (13–30 Hz). The absolute powers were then averaged over all time windows for each frequency band and subsequently ln-transformed for further statistical analysis. We examined the absolute power at four regions by averaging the power at corresponding electrodes: frontal (F3, F4, Fz), central (C3, C4, Cz), parietal (P3, P4, Pz) and occipital (O1, O2).

### Statistical analysis

Statistical analyses were conducted using SPSS Statistics 24.0 (IBM corp.; Armonk, New York). Subjects were split into stable (averaged stability score = 8.89, SD = 1.37) and unstable (averaged stability score = 3.08, SD = 1.04) groups based on the median of the arousal stability score (median = 6). Measures of differences between unstable and stable groups were conducted with Independent Sample T-Test for metric and Pearson Chi Square (X^2^) Test for nominal variables, respectively. Since the arousal stability score was nominally scaled after median split, we ran Eta correlation to determine the strength of nonlinear association between arousal stability score and self-reported measures as well as the absolute powers in each frequency band Eta-squared (Eta^2^) was given to specify how much variation could be explained by the arousal stability score. Cohen’s d was provided to evaluate the effect sizes for comparisons of EEG absolute powers between groups. For all analyses statistical significance was set at p < 0.05.

## Results

### Sample description

The main demographic characteristics are summarized in Table 2. No differences in age, gender, marital status, graduation, habituated sleep duration and use of alcohol were observed (0.161 ≤ *p* < 1.000) between unstable and stable groups. However, there were significantly more smokers (*p *= 0.004) in the stable than in the unstable group. Considering the possible effect of nicotine use on the EEG arising from Knott [46], we did an extra analysis using smokers and nonsmokers as group variable to test the impact of nicotine use on the absolute EEG powers. There were totally 17 smokers, 13 nonsmokers and 1 with missing information in our sample, between smokers and nonsmokers, no differences could be obtained for delta (degree of freedom [df] = 28 for all comparisons; frontal: T = − 0.717, p = 0.479; central: T = − 0.943, p = 0.354; parietal: T = − 1.185, p = 0.246; occipital: T = − 0.407, p = 0.687), theta (frontal: df = 18.572, T = − 0.492, p = 0.628; central: df = 28, T = − 0.991, p = 0.330; parietal: df = 28, T = − 1.049, p = 0.303; occipital: df = 17.702, T = − 0.738, p = 0.470), alpha (df = 28 for all comparisons; frontal: T = − 0.826, p = 0.416; central: T = − 0.801, p = 0.430; parietal: T = − 0.979, p = 0.336; occipital: T = − 0.732, p = 0.470) and beta activity (df = 28 for all comparisons; frontal: T = 0.060, p = 0.953; central: T = − 0.210, p = 0.835; parietal: T = − 0.443, p = 0.661; occipital: T = − 0.434, p = 0.667) at any sites.

**Table 2:** Differences in demographic and clinic characteristics and self-report measures between unstable and stable groups of ADHD patients

	Unstable group		Stable group		Test	*p*
	Mean (SD)	n/available n	Mean (SD)	n/available n		
Number		13		18		
Gender (women)^a^	46.15	6/13	44.44	8/18	X^2^ = 0.009	1.000
Age (in years)	32.23 (7.98)	13	32.28 (6.41)	18	T = − 0.729	0.435
Alcohol users^a^	83.30	10/12	88.89	16/18	X^2^ = 0.192	0.661
Nicotine users^a^	25.00	3/12	77.78	14/18	X^2^ = 8.167*	0.004
General sleep	7.45 (2.87)	11	7.23 (2.64)	17	T = 0.208	0.837
Marital status						
Single	92.31	12/13	83.33	15/18	X^2^ = 3.621	0.164
Married	0.00	0/13	16.67	3/18		
Divorced	7.69	1/13	0.00	0/18		
Graduation						
9th class	30.77	4/13	16.67	3/18	X^2^ = 3.046	0.218
10th class	7.69	1/13	33.33	6/18		
12th class	61.54	8/13	50.00	9/18		
CAARS (%)^b^						
DSM-IA	76.92	10/13	88.24	15/17	X^2^ = 0.679	0.410
DSM-HYI	61.54	8/13	52.94	9/17	X^2^ = 0.222	0.638
DSM-G	76.92	10/13	64.71	11/17	X^2^ = 0.524	0.469
CAARS (T)						
DSM-IA	79.00 (13.90)	13	78.94 (9.12)	17	T = 0.014	0.989
DSM-HYI	70.69 (14.79)	13	68.53 (13.75)	17	T = 0.413	0.683
DSM-G	77.92 (13.08)	13	76.35 (10.70)	17	T = 0.362	0.720
WURSK	42.92 (14.08)	13	36.94 (11.71)	17	T = 1.270	0.214
ASRS						
A-cutoff	4.46 (1.94)	13	4.52 (1.42)	17	T = − 0.111	0.913
A-sum	17.38 (3.62)	13	16.71 (2.64)	17	T = 0.595	0.557
B-cutoff	9.08 (2.60)	13	9.23 (1.82)	17	T = − 0.197	0.846
B-sum	32.31 (6.97)	13	32.83 (5.49)	17	T = − 0.227	0.882
BDI (%)^c^	33.33	4/12	70.59	12/17	X^2^ = 5.855*	0.016
BDI (score)	9.75 (5.63)	12	19.06 (11.82)	17	T = − 2.802*	0.018

*T* T scores, *BDI* Beck Depression Inventory

* p < 0.05

^a^Percentage of participants in this group

^b^Percentage of patients scoring above T-value of 70 in each subscale of CAARS

^c^Percentage of patients scoring above 13 in BDI

**Fig. 1:**
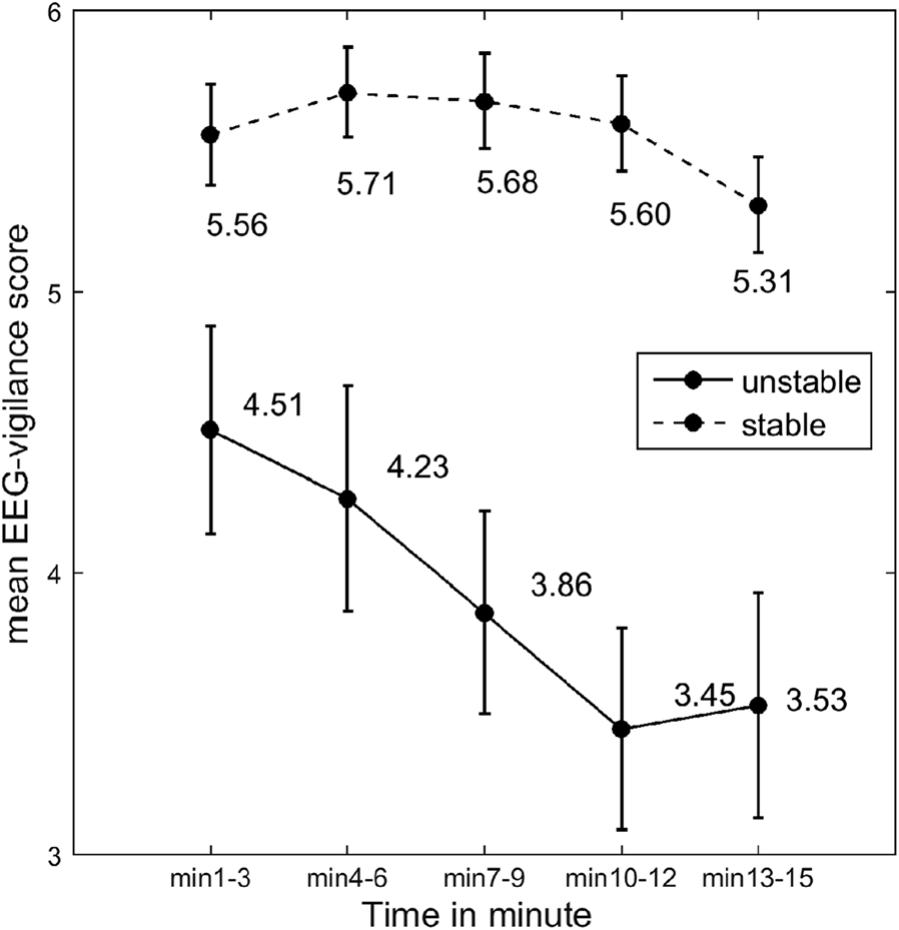
Mean vigilance level in 3-min time blocks in unstable and stable groups during resting EEG. Unstable refers to adult attention-deficit/hyperactivity disorder (ADHD) patients having an arousal stability score below median (= 6), while stable indicates adult ADHD patients having an arousal stability score above the median. The data points are shown as the mean ± 1 standard error

The change of mean EEG-vigilance score (indicated the mean EEG-vigilance level) over time is presented in Fig. 1, indicating a distinct separation between unstable and stable groups.

### Differences in self-report measures

No significant group differences in CAARS, WURS-K or ASRS were found (0.214 ≤ *p* ≤ 0.989). Detailed results are presented in Table 2.

Significantly more ADHD patients in the stable group reported depressive symptoms compared to those in unstable group (33.33% vs. 70.59%, *p *= 0.016). In line with this, a significantly higher mean BDI score (*p *= 0.018) was found in the stable group.

### Correlation of self-reported measures and absolute EEG powers

All correlation coefficients and their effect sizes are summarized in Table 3. Concerning the relationship between arousal stability score and depressive symptoms in adult ADHD patients, we found a significant positive correlation between arousal stability and BDI scores (*p *= 0.018). About 19% variation in BDI score can be explained by the arousal stability score. Significant negative correlations were also obtained between the arousal stability score and absolute delta power at all regions (0.001 ≤ *p* ≤ 0.033) and absolute theta power at central (*p *= 0.032) and occipital (*p *= 0.021) regions. The arousal stability score explained about 15% to 30% of overall variations in the absolute delta power, while about 12% to 17% of variations in absolute theta power can be explained by stability score.

**Table 3:** Results of nonlinear correlations between categorical arousal stability score (unstable or stable) and self-report measures and absolute EEG powers

	Arousal stability score (unstable or stable)			
	Eta	*p*	Eta^2^	Available n
CAARS^a^				
DSM-IA	− 0.003	0.989	9E−6	31
DSM-HYI	− 0.078	0.683	0.006	31
DSM-G	− 0.068	0.720	0.004	31
WURSK	− 0.233	0.214	0.054	31
ASRS				
A-cutoff	0.021	0.913	4E−4	31
A-sum	− 0.112	0.557	0.013	31
B-cutoff	0.037	0.846	0.001	31
B-sum	0.043	0.822	0.002	31
BDI	0.437*	0.018	0.191	29
Delta				
F	− 0.548*	0.001	0.300	31
C	− 0.436*	0.014	0.190	31
P	− 0.532*	0.002	0.283	31
O	− 0.383*	0.033	0.147	31
Theta				
F	− 0.352	0.052	0.124	31
C	− 0.385*	0.032	0.148	31
P	− 0.290	0.114	0.084	31
O	− 0.413*	0.021	0.171	31
Alpha				
F	− 0.019	0.921	3E−4	31
C	0.002	0.992	4E−6	31
P	0.068	0.714	0.005	31
O	− 0.034	0.857	0.001	31
Beta				
F	− 0.190	0.307	0.036	31
C	− 0.228	0.217	0.052	31
P	− 0.021	0.910	4E−4	31
O	− 0.102	0.585	0.010	31

*BDI* Beck Depression Inventory, *F* frontal lobe, *C* central lobe, *P* parietal lobe, *O* occipital lobe

*p < 0.05

^a^T scores in CAARS subscale

### Comparison of absolute EEG powers between groups

**Fig. 2:**
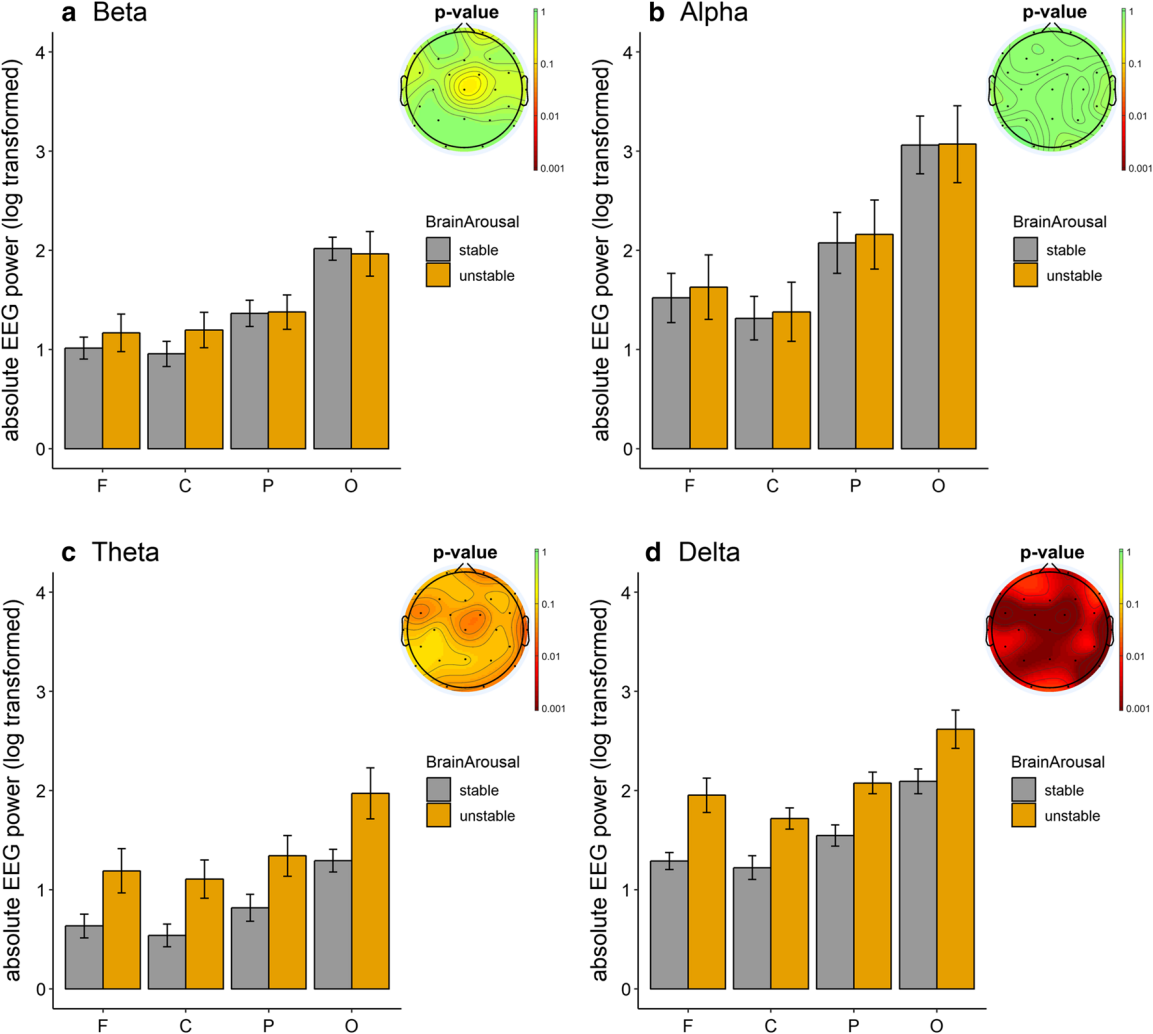
Absolute electroencephalogram (EEG) powers in unstable and stable group and their difference mappings. The absolute EEG powers in corresponding frequency bands, i.e. **a** beta, **b** alpha, **c** theta and **d** delta were log-transformed. Unstable refers to adult attention-deficit/hyperactivity disorder (ADHD) patients having an arousal stability score below median (= 6), while stable indicates adult ADHD patients having arousal stability score above the median. The data is shown as the mean ± 1 standard error. Brain maps (p-value) showing statistical difference between unstable and stable group. ADHD patients in stable group had significantly reduced absolute delta and theta power at all mentioned regions as compared to those in the unstable group. *F* frontal site, *C* central site, *P* parietal site, *O* occipital site

Figure 2 shows absolute EEG powers in beta, alpha, theta and delta bands in each group at respective frontal, central, parietal and occipital regions, and the corresponding topographical mapping of differences of absolute powers between unstable and stable groups. Independent sample T-tests revealed null group effect on *absolute beta power* at any site (frontal: df = 29, T = 0.740, *p *= 0.465, *d *= 0.370; central: df = 29, T = 1.139, *p *= 0.264, *d *= 0.454; parietal: df = 29, T = 0.066, *p *= 0.948, *d *= 0.042; occipital: df = 29, T =  −0.229, *p *= 0.820, *d *= 0.190). This was the same for *absolute alpha power* at all regions (frontal: df = 29, T = 0.279, *p *= 0.782, *d *= 0.037; central: df = 29, T = 0.187, *p *= 0.853, *d *= 0.004; parietal: df = 29, T = 0.970, *p *= 0.858, *d *= 0.141; occipital: df = 23.890, T = 0.019, *p *= 0.985, *d *= 0.066). However, higher *absolute theta power* was found in the unstable group at frontal (df = 29, T = 2.344, *p *= 0.026, *Cohen’s d *= 0.682), central (df = 29, T = 2.700, *p *= 0.011, *d *= 0.772), parietal (df = 29, T = 2.219, *p *= 0.034, *d *= 0.573) and occipital region (df = 16.776, T = 2.408, *p *= 0.028, *d *= 0.817) compared to the stable group. The comparisons also reached significance levels for *absolute delta power* at all regions (frontal: df = 17.795, T = 3.455, *p *= 0.013, *d *= 1.186; central: df = 29, T = 3.076, *p *= 0.005, *d *= 0.936; parietal: df = 29, T = 3.415, *p *= 0.002, *d *= 1.209; occipital: df = 29, T = 2.371, *p *= 0.025, *d *= 0.767).

## Discussion

The main purpose of this study was to find out whether the stable arousal regulation in adult ADHD patients relates to depressive symptomology. Supporting our hypothesis, we found an association between brain arousal stability, as indexed by the arousal stability score measured in a resting-state-EEG, and depressive symptom severity, as indexed by the BDI score, in adult patients diagnosed with ADHD. In this study, arousal stability score did not associate with self-evaluations concerning ADHD symptoms (see Table 3), suggesting that the severity of ADHD symptoms did not correlate with brain arousal regulation in our sample. However, the brain arousal state, as expected, positively related to BDI scores (see Table 3) -the higher the arousal stability, the greater was the depressive symptom severity. This was also supported by significant group (unstable vs. stable group split by median of arousal stability score) differences in depressive symptom severity as assessed by BDI score, but not in ADHD related symptoms (see Table 2). These findings support and extend the brain arousal regulation model by showing the relationship of stable arousal regulation in ADHD patients who are generally supposed having unstable arousal regulation to depressive symptoms.

Regarding the extensive evidence for critical role of the LC-NE system in the modulation of cortical arousal that are reflected in EEG signals [28–30], these findings are also consistent with the assumption of the balance of the tonic and phasic activity of the LC-NE system in ADHD raised by Howells et al. [35]. These authors suggested that ADHD is a model of hypoarousal resulting from decreased tonic firing of the LC-NE system. In demand of e.g. acute stressors, a phasic activation of the LC-NE system would be achieved under the requirement of performance compensation. We suspect that during this period, the regulation of brain arousal in adult ADHD is supposed to be more stable or even hyperstable. The depressive symptom is therefore a result of disrupted tonic and phasic LC-NE activity. To test this model furthers studies with repeated session are necessary.

Furthermore, the impact of brain arousal regulation on cortical activities measured by quantitative EEG can be demonstrated by the reduced absolute powers in delta and theta bands (see middle panel in Fig. 1) at ADHD patients in the stable group. They correspondingly exhibited more stable brain arousal regulation, i.e. little change in EEG-vigilance from high to low stages over the entire recording period, as compared to those patients in the unstable group showing a clear decline in EEG-vigilance over time (see Fig. 1). In line with these results, reduced slow activities, i.e. delta activity, has been consistently found in MDD patients [47–53]. Additionally, we found negative links between arousal stability and absolute delta powers at all sites (see Table 3). Based on these results, we suggest that the hyperstable arousal regulation in ADHD patients with MDD is possibly linked to the reduction in delta power.

However, conflicting results have also been reported in several work groups showing increased delta/theta activities in patients with MDD only compared to healthy controls [54, 55], or null effect between ADHD patients with and without depressive symptoms [9]. This inconsistency might depend on the group dividing method we used for our sample: we divided groups based on the median of arousal stability score, where EEG-vigilance stage B2/3 (i.e. indicating appearance of theta/delta activities) mainly occurred already during the first 5 min (see Table 1) of the recording session, whereas the quantitative EEG is a measure without consideration of time varying effect on the EEG powers. Thus, the absolute EEG powers presented in this study were general markers for EEG power over a long period recording time and did not show any temporal characteristics. This is also a suitable explanation for the lack of observed between-group differences in alpha activities.

Given the obvious different amounts of smokers in stable and unstable group as well as the effect of life-long smoking on the EEG [46], we additionally tested the impact of role of smoking on the absolute EEG activities. However, this is not the case in our sample. No differences between smokers and nonsmokers for any bands or at any sites could be determined. The effect of life-long smoking on the EEG is difficult separate from the influence attributed to normal aging. The neurophysiological effects of smoking are often attributed to nicotine. However, this has already been questioned by studies showing changes in the EEG caused by zero nicotine cigarette smoking [56]. This leads to a speculation that the registered changes in EEG might be caused by other substance or there is additional process mediating between smoke and the EEG oscillations.

Some limitations, such as small sample size and absence of a healthy control group, should be mentioned for this study and further studied. The depressive symptoms in this study were indicated by self-reports, a diagnosis of MDD meeting DSM criteria should be considered in future studies.

## Conclusions

Overall, the results from this study demonstrate the association between brain arousal regulation as assessed by the stability score using EEG in resting state and depressive symptom severity as indexed by BDI scores in adult patients with ADHD. This association has been further supported by the effect of brain arousal regulation on absolute power in different frequency bands utilizing quantitative EEG analysis. In view of the high comorbidity of depression and ADHD and the often difficult clinical differentiation of both diseases, brain arousal regulation could be a clinical marker of differentiation, which should be further evaluated in larger studies. The differentiation between ADHD and depression is not only important diagnostically, but also important for the selection of the right treatment (psychostimulants vs. antidepressants). Based on the brain arousal regulation model of affective disorders and ADHD [18, 23], it has to be assumed that psychostimulants have a stabilizing effect on brain arousal regulation, so that their use in patients with comorbid MDD could lead to a worsening of the depressive symptoms at least. This is also in consensus with the generally accepted recommendations for the treatment of comorbid depression and ADHD: for severe depressive symptoms, guideline-based treatment for depression is generally recommended [6, 7]. Further studies should also address the question of whether ADHD patients with MDD are less likely to respond to treatment with psychostimulants.

## Data Availability

The datasets supporting the conclusions of this article are included within the article. The spreadsheets and corresponding syntax are available on request from the first author.

## Abbreviations

ADHD: attention-deficit/hyperactivity disorder
MDD: major depression disorder
EEG: electroencephalography
LC-NE system: locus coeruleus-norepinephrine system
VIGALL: Vigilance Algorithm Leipzig
DSM-IV: diagnostic and statistical manual of mental disorder 4th edition
CAARS: Conners’ Adults ADHD rating scale-self
DSM-IA: DSM-inattention
DSM-HYI: DSM-hyperactivity/impulsivity
DSM-G: DSM-global
ASRS: Adult ADHD self-report scale
WURS-K: Wender Utah rating scale
BDI: Beck Depression Inventory
ICA: independent component analysis
FFT: fast Fourier transformation
LORETA: low resolution electromagnetic tomography
SEM: slow eye movement

## References

[1] Simon V, Czobor P, Bálint S, Mészáros Á, Bitter I (2009). Prevalence and correlates of adult attention-deficit hyperactivity disorder: meta-analysis. Br J Psychiatry.

[2] Miesch M, Deister A (2018). Attention-deficit/hyperactivity disorder (ADHD) in adult psychiatry: data on 12-month prevalence, risk factors and comorbidity. Fortschr Neurol Psychiatr.

[3] Fischer AG, Bau CH, Grevet EH, Salgado CA, Victor MM, Kalil KL, Sousa NO, Garcia CR, Belmonte-de-Abreu P (2007). The role of comorbid major depressive disorder in the clinical presentation of adult ADHD. J Psychiatr Res.

[4] Bond DJ, Hadjipavlou G, Lam RW, Mclntyre RE, Beaulieu S, Schaffer A, Weiss M, Canadian Network for Mood and Anxiety Treatments (CANMAT) Task Force (2012). The Canadian Network for Mood and Anxiety Treatments (CANMAT) task force recommendations for the management of patients with mood disorders and comorbid attention-deficit/hyperactivity disorder. Ann Clin Psychiatry..

[5] Sawyer MG, Reece CE, Sawyer AC, Johnson S, Lawrence D, Zubrick SR (2017). The prevalence of stimulant and antidepressant use by australian children and adolescents with attention-deficit/hyperactivity disorder and major depressive disorder: a National Survey. J Child Adolesc Psychopharmacol..

[6] Hauck TS, Lau C, Wing LLF, Kurdyak P, Kurdyak P, Tu K (2017). ADHD treatment in primary care: demographic factors, medication factors, medication trends, and treatment predictors. Can J Psychiatry.

[7] Kooij SJ, Bejerot S, Blackwell A, Caci H, Casas-Brugue M, Carpentier PJ, Edvinsson D, Fayyad J, Foeken K, Fitzgerald M, Gaillac V, Ginsberg Y, Henry C, Krause J, Lensing MB, Manor I, Niederhofer H, Nunes-Filipe C, Ohlmeier MD, Oswald P, Pallanti S, Pehlivanidis A, Ramos-Quiroga JA, Rastam M, Ryffel-Rawak D, Stes S, Asherson P (2010). European consensus statement on diagnosis and treatment of adult ADHD: the European Network Adult ADHD. BMC Psychiatry..

[8] Satterfield JH, Dawson ME (1971). Electrodermal correlates of hyperactivity in children. Psychophysiology.

[9] Barry RJ, Clarke AR, Selikowitz M, MacDonald B, Dupuy FE (2012). Caffeine effects on resting-state electrodermal levels in AD/HD suggest an anomalous arousal mechanism. Biol Psychol.

[10] Broyd SJ, Johnstone SJ, Barry RJ, Clarke AR, McCarthy R, Selikowitz M, Lawrence CA (2005). The effect of methylphenidate on response inhibition and the event-related potential of children with attention deficit/hyperactivity disorder. Int J Psychophysiol.

[11] Conzelmann A, Gerdes AB, Mucha RF, Weyers P, Lesch KP, Bahne CG, Fallgatter AJ, Renner TJ, Warnke A, Romanos M, Pauli P (2014). Autonomic hypoactivity in boys with attention-deficit/hyperactivity disorder and the influence of methylphenidate. World J Biol Psychiatry..

[12] Hermens DF, Williams LM, Lazzaro I, Whitmont S, Melkonian D, Gordon E (2004). Sex differences in adult ADHD: a double dissociation in brain activity and autonomic arousal. Biol Psychol.

[13] Lazzaro I, Gordon E, Li W, Lim CL, Plahn M, Whitmont S, Clarke S, Barry RJ, Dosen A, Meares R (1999). Simultaneous EEG and EDA measures in adolescent attention deficit hyperactivity disorder. Int J Psychophysiol.

[14] Barry RJ, Clarke AR, Johnstone SJ (2003). A review of electrophysiology in attention-deficit/hyperactivity disorder: I. Qualitative and quantitative electroencephalography. Clin Neurophysiol..

[15] Barry RJ, Clarke AR, Johnstone SJ, McCarthy R, Selikowitz M (2009). Electroencephalogram theta/beta ratio and arousal in attention-deficit/hyperactivity disorder: evidence of independent processes. Biol Psychiatry.

[16] Kim WJ, Kim SY, Choi J, Kim KM, Nam SH, Min KJ, Lee YS, Choi TY (2017). Differences in resting-state quantitative electroencephalography patterns in attention deficit/hyperactivity disorder with or without comorbid symptoms. Clin Psychopharmacol Neurosci..

[17] Loo SK, Cho A, Hale TS, McGough J, McCracken J, Smmalley SL (2013). Characterization of the theta to beta ratio in ADHD: identifying potential sources of heterogeneity. J Atten Disord..

[18] Hegerl U, Hensch T (2014). The vigilance regulation model of affective disorders and ADHD. Neurosci Biobehav Rev.

[19] Hegerl U, Himmerich H, Engmann B, Hensch T (2010). Mania and attention-deficit/hyperactivity disorder: common symptomatology, common pathophysiology and common treatment?. Curr Opin Psychiatry..

[20] Huang J, Hensch T, Ulke C, Sander C, Spada J, Jawinski P, Hegerl U, Hensch T (2017). Evoked potentials and behavioral performance during different states of brain arousal. BMC Neurosci..

[21] Huang J, Ulke C, Sander C, Jawinski P, Spada J, Hegerl U, Hensch T (2018). Impact of brain arousal and time-on-task on autonomic nervous system activity in the wake-sleep transition. BMC Neurosci..

[22] Ulke C, Huang J, Schwabedal JTC, Surova G, Mergl R, Hensch T (2017). Coupling and dynamics of cortical and autonomic signals are linked to central inhibition during the wake-sleep transition. Sci Rep..

[23] Strauss M, Ulke C, Paucke M, Huang J, Sander C, Stark T, Hegerl U (2018). Brain arousal regulation in adults with attention-deficit/hyperactivity disorder (ADHD). Psychiatry Res.

[24] Hegerl U, Wilk K, Olbrich S, Schoenknecht P, Sander C (2012). Hyperstable regulation of vigilance in patients with major depression disorder. World J Biol Psychiatry..

[25] Schmidt FM, Sander C, Dietz ME, Nowak C, Schroeder T, Mergl R, Schoenknecht P, Himmerich H, Hegerl U (2017). Brain arousal regulation as response predictor for antidepressant therapy in major depression. Sci Rep..

[26] Ulke C, Tenke CE, Kayser J, Sander C, Böttger D, Wong LYX, Alvarenga JE, Fava M, McGrath PJ, Deldin PJ, Mcinnis MG, Trivedi MH, Weissman MM, Pizzagalli DA, Hegerl U, Bruder GE (2019). Resting EEG measure of brain arousal in a multisite study of major depression. Clin EEG Neurosci.

[27] Ulke C, Wittekind DA, Spada J, Franik K, Jawinski P, Hensch T, Hegerl U (2019). Brain arousal regulation in SSRI-medicated patients with major depression. J Psychiatr Res.

[28] Foote SL, Bloom FE, Aston-Jones G (1983). Nucleus locus ceruleus: new evidence of anatomical and physiological specificity. Physiol Rev.

[29] Foote SL, Moorison JH (1987). Extrathalamic modulation of cortical function. Annu Rev Neurosci.

[30] Aston-Jones G, Bloom FE (1981). Activity of norepinephrine-containing locus coeruleus neurons in behaving rats anticipates fluctuations in the sleep-waking cycle. J Neurosci.

[31] Alexander DM, Hermens DF, Keage HA, Clark CR, Williams LM, Kohn MR, Clarke SD, Lamb C, Gordon E (2008). Event-related wave activity in the EEG provides new marker of ADHD. Clin Neurophysiol.

[32] Benarroch EE (2009). The locus ceruleus norepinephrine system: functional organization and potential clinical significance. Neurology..

[33] Ding YS, Naganawa M, Gallezot JD, Nabulsi N, Lin SF, Ropchan J, Weinzimmer D, MaCarthy TJ, Carson RE, Huang Y, Laruelle M (2014). Clinical does of atomoxetine significantly occupy both norepinephrine and serotonin transports: implications on treatment of depression and ADHD. Neuroimage..

[34] Viggiano D, Ruocco LA, Arcieri S, Sadile AG (2004). Involvement of norepinephrine in the control of activity and attentive processes in animal models of attention deficit hyperactivity disorder. Neural Plasst..

[35] Howells FM, Stein DJ, Russell VA (2012). Synergistic tonic and phasic activity of the locus coeruleus norepinephrine (LC-NE) arousal system is required for optimal attentional performance. Metab Brain Dis.

[36] Weinstein D, Staffelbach D, Biaggio M (2000). Attention-deficit hyperactivity disorder and posttraumatic stress disorder: differential diagnosis in childhood sexual abuse. Clin Psychol Rev..

[37] Hegerl U, Sander C, Ulke C, Böttger D, Hensch T, Huang J, Mauche N, Olbrich S. Vigilance Algorithm Leipzig (VIGALL) 2.1 Manual; 2017. https://github.com/danielboettger/VIGALL. Accessed 19 Aug 2019.

[38] Ulke C, Sander C, Jawinski P, Mauche N, Huang J, Spada J, Wittekind D, Mergl R, Luck T, Riedel-Heller S, Hensch T, Hegerl U (2017). Sleep disturbance and upregulation of brain arousal during daytime in depressed versus non-depressed elderly subjects. World J Biol Psychiatry..

[39] Jawinski P, Kittel J, Huang J, Spada J, Ulke C, Wirkner K, Hensch T, Hegerl U (2017). Recorded and reported sleepiness: the association between brain arousal in resting state and subjective daytime sleepiness. Sleep..

[40] Olbrich S, Mulert C, Karch S, Trenner M, Leicht G, Pogarell O, Hegerl U (2009). EEG-vigilance and BOLD effect during simultaneous EEG/fMRI measurement. Neuroimage..

[41] Olbrich S, Sander C, Matschinger H, Mergl R, Trenner M, Schoenknecht P, Hegerl U (2011). Brain and body: associations between EEG-vigilance and the autonomous nervous system activity during rest. J Psychophysiol..

[42] Christiansen H, Hirsch O, Abdel-Hamid M, Bernhard K (2014). Conners Skalen zu Aufmerksamkeit und Verhalten für Erwachsene (CAARS). Deutschsprachige Adaptation der Conners’ Adult ADHD Rating Scale (CAARS) von C. Keith Conners, Drew Erhard und Elizabeth Sparrow.

[43] Kessler RC, Adler L, Ames M, Demler O, Faraone S, Hiripi E, Howes MJ, Jin R, Secnik K, Spencer T, Ustun TB, Walters EE (2005). The World Health Organization Adult ADHD Self-Report Scale (ASRS): a short screening scale for use in the general population. Psychol Med.

[44] Retz-Junginger P, Retz W, Blocher D, Weijers HG, Trott GE, Wender PH, Roessler M (2002). Wender Utah Rating Scale (WURS-k) Die deutsche Kurzform zur retrospektiven Erfassung des hyperkinetischen Syndroms bei Erwachsenen. Nervenarzt..

[45] Beck AT, Steer RA, Brown GK (1996). Beck-Depressions-Inventory–BDI.

[46] Knott VJ (2001). Electroencephalographic characterization of cigarette smoking behavior. Alchhol..

[47] Armitage R (1995). Microarchitectural findings in sleep EEG in depression: diagnostic implications. Biol Psychiatry.

[48] Armitage R, Hoffmann RF, Fitch T, Trivedi MH, Rush AJ (2000). Temporal characteristics of delta activity during NREM sleep in depressed outpatients and healthy adults: group and sex effects. Sleep.

[49] Armitage R, Hoffmann RF, Trivedi MH, Rush AJ (2000). Slow-wave activity in NREM sleep: sex and age effects in depressed outpatients and healthy controls. Psychiatry Res.

[50] Cheng P, Goldschmied J, Deldin P, Hoffmann R, Armitage R (2015). The role of fast and slow EEG activity during sleep in males and females with major depressive disorder. Psychophysiology.

[51] Goldschmied JR, Cheng P, Armitage R, Deldin PJ (2014). Examining the effects of sleep delay on depressed males and females and healthy controls. J Sleep Res.

[52] Kupfer DJ, Grochocinski VJ, McEachran AB (1986). Relationship of awakening and delta sleep in depression. Psychiatry Res.

[53] Lotrich FE, Germain A (2015). Decreased delta sleep ratio and elevated alpha power predict vulnerability to depression during interferon-alpha treatment. Acta Neuropsychiatr..

[54] Howells FM, Temmingh HS, Hsieh JH, van Dijen AV, Baldwin DS, Stein DJ (2018). Electroencephalographic delta/alpha frequency activity differentiates psychotic disorders: a study of schizophrenia, bipolar disorder and methamphetamine-induced psychotic disorder. Transl Psychiatry..

[55] Tesler N, Gerstenberg M, Franscini M, Jenni OG, Walitza S, Huber R (2015). Increased frontal sleep slow wave activity in adolescents with major depression. NeuroImage Clin..

[56] Domino EF, Matsuoka S (1994). Effects of tobacco smoking on the topographic EEG-I. Prog Neuropsychopharmacol Biol Psychiatry.

